# Analysis of Traffic Crashes Caused by Motorcyclists Running Red Lights in Guangdong Province of China

**DOI:** 10.3390/ijerph18020553

**Published:** 2021-01-11

**Authors:** Guangnan Zhang, Ying Tan, Qiaoting Zhong, Ruwei Hu

**Affiliations:** 1 Center for Studies of Hong Kong, Macao and Pearl River Delta, Institute of Guangdong, Hong Kong and Macao Development Studies, Sun Yat-Sen University, Guangzhou 510275, China; zhgnan@mail.sysu.edu.cn; 2 School of Economics and Trade, Guangdong University of Finance, Guangzhou 510521, China; yingtan.cn@hotmail.com; 3 School of Public Health, Sun Yat-Sen University, Guangzhou 510080, China; huruwei@mail.sysu.edu.cn

**Keywords:** traffic violation, injury severity, road safety, risk factor, motorcyclist

## Abstract

Motorcycles are among the primary means of transport in China, and the phenomenon of motorcyclists running red lights is becoming increasingly prevalent. Based on the traffic crash data for 2006–2010 in Guangdong Province, China, fixed- and random-parameter logit models are used to study the characteristics of motorcyclists, vehicles, roads, and environments involved in red light violations and injury severity resulting from motorcyclists’ running red lights in China. Certain factors that affect the probability of motorcyclists running red lights are identified. For instance, while the likelihood of violating red light signals during dark conditions is lower than during light conditions for both car drivers and pedestrians, motorcyclists have significantly increased probability of a red light violation during dark conditions. For the resulting severe casualties in red-light-running crashes, poor visibility is a common risk factor for motorcyclists and car drivers experiencing severe injury. Regarding the relationship between red light violations and the severity of injuries in crashes caused by motorcyclists running red lights, this study indicated that driving direction and time period have inconsistent effects on the probability of red light violations and the severity of injuries. On the one hand, the likelihood of red light violations when a motorcycle rider is turning left/right is higher than when going straight, but this turning factor has a nonsignificant impact on the severity of injuries; on the other hand, reversing, making a U-turn and changing lanes have nonsignificant effects on the probability of motorcyclists’ red light violations in contrast to going straight, but have a very significant impact on the severity of injuries. Moreover, the likelihood of red light violations during the early morning is higher than off-peak hours, but this time factor has a negative impact on the severity of injuries. Measures including road safety educational programs for targeted groups and focused enforcement of traffic policy and regulations are suggested to reduce the number of crashes and the severity of injuries resulting from motorcyclists running red lights.

## 1. Introduction

Running red lights is a major cause of crashes at intersections, posing a higher risk of injury than other kinds of traffic violations [1,2]. The AAA Foundation for Traffic Safety has revealed that red-light running (RLR) deaths in the United States hit a 10-year high in 2017 and 28% of crash deaths that occurred at signalized intersections were the result of a driver running through a red light. More than 184 drivers daily were caught failing to stop at red lights in the United Kingdom in 2015 (more details can be found at https://www.thisismoney.co.uk/money/cars/article-3589194/The-roads-drivers-caught-running-red-lights-revealed.html.). Among all police-reported crashes in 2015 in Thailand, 1.96% were caused by drivers violating the red light signal [3]. According to the statistics revealed by the Ministry of Public Security, 4227 severe-injury crashes and 789 fatalities between January and October 2012 in China were attributed to RLR [4].

Previous studies have reported various rates of running red lights among road users. For instance, a study by Yan et al. [5] showed that the RLR rate in Changsha, China was 0.14% for motorists, which was far lower than those for motorcyclists (18.64%), bicyclists (18.74%) and pedestrians (18.54%). Kim et al. [6] compared the odds of running red lights between drivers and pedestrians in Hawaii, United States and concluded that drivers tend to commit proportionately more RLR than pedestrians.

It is critical to identify the influencing factors leading to RLR behavior for different road users in different contexts [5]. However, research efforts have focused on car drivers, e-bike riders, cyclists and pedestrians rather than motorcycle riders, who dominate traffic in developing countries such as China. Not only the riders of motorcycles but also the use of motorcycles in China are common, making the problem of motorcyclists running red lights more prominent in China than in other countries. Most of the motorcyclists in China have not undergone formal training on how to ride a motorcycle, and riding without number plates is quite common [7]. In addition, motorcycles in China are mainly used for delivering food and goods, services with a high demand for efficiency, resulting in many motorcyclists choosing to run red lights or violating traffic safety laws to complete their tasks on time. By assessing the impact of various risk factors on motorcyclists’ RLR violations and related accident severity in Guangdong Province of China, results arising from this study will shed lights on the development of similar (adjusted) measures to reduce the number of crashes and the severity of injuries resulting from motorcyclists running red lights, and to promote road safety in other regions.

Based on previous limited studies, there are few contributing factors affecting motorcyclists’ RLR violations, such as human characteristics, driving conditions, and driving environment. For human characteristics, both Chen et al. [8] and Jensupakarn and Kanitpong [9] revealed that male and young motorcycle riders were more likely to commit RLR violations. Motorcyclists whose occupation is a business person/trader and students are more likely to run red lights than other occupations [9]. 

The approaching speed and the direction of travel of motorcycles, helmet use, the presence of a pillion passenger, and the distance from the subject to the stop line all significantly affect RLR violations. Evidence shows that motorcycle riders carrying pillion passengers are less likely to execute RLR violations [8,9], which could be explained by the ease and stability of riding without a passenger, or a sense of responsibility for the passenger’s safety [8]. While the approaching speed had a negative effect on RLR rates in Jensupakarn and Kanitpong’s study [9] on motorcyclists in Thailand, Chen et al. [8] found that motorcyclists travelling at higher speeds were more likely to commit RLR violations in Taiwan. Jensupakarn and Kanitpong’s research [9] also suggested that motorcyclists riding straight through an intersection are less likely to run a red light compared to when they have to make a turn, and motorcycle riders who do not wear a helmet tend to commit RLR violations. Finally, motorcycle riders are less likely to run a red light if they are further from a junction when the light turns red [8]. 

With regard to the driving environment, evidence shows that the likelihood of motorcyclists running a red light is higher at night time [8,9], in periods with lower traffic volume [8] and during off-peak hours [5,8], but is lower on a weekend or a holiday [5].

Despite the increasing prevalence of motorcyclists running red lights in China, the relevant literature is limited. Only a study by Yan et al. [5] examined the relationship between RLR violation rates and the day (weekend or work day) and time period for motorcyclists in Changsha, China. Although such observational investigation is extremely valuable, it is costly to undertake by its very nature, and thus is performed on selected intersections. Even if the estimates obtained are reliable, they can only reflect some specific circumstances at a particular time and place; therefore, sampling errors may arise, and many important variables (such as individual characteristics of motorcycle riders) may be lost. More importantly, the extent to which the results arising from observation surveys are broadly generalizable is unclear [10], leading to a lack of comprehensiveness in the analyses of risk factors in red light violations and the severity of injuries caused by motorcyclists. With this in mind, the specific objectives of this study were to identify the risk factors related to personal characteristics, vehicle characteristics, road conditions and environmental conditions affecting (1) motorcycle riders’ RLR violations and (2) the severity of injuries caused by motorcycle RLR crashes, using data from the road traffic crash database of China’s Public Security Department from 2006 to 2010 in the Guangdong Province of China.

## 2. Materials and Methods

### 2.1. Data

The data used in this study, obtained from the Guangdong Provincial Security Department, were extracted from the Traffic Management Sector-Specific Incident Case Data Report. The data were recorded and reported by the traffic police who conducted on-scene assessments and provided feedback within 24 h to the headquarters of the Traffic Management Department. The information was recorded according to the Code of Traffic Crash Information issued by the Computer and Information Processing Standardization Commission under the Security Department of the country. Each sample included detailed indexes about the characteristics of drivers/riders, injury severity, vehicle features, road conditions, crash time, as well as environmental conditions, such as the level, form, and cause of the crash [2]. 

**Figure 1:**
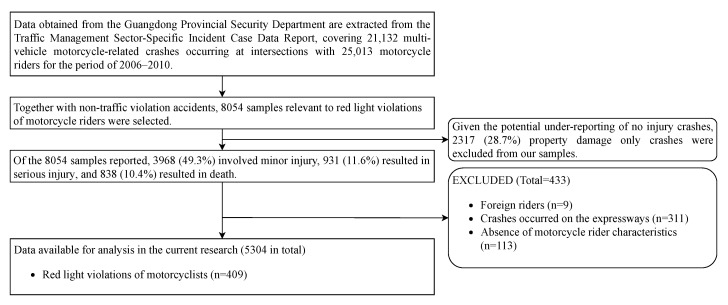
Data flow diagram for analysis of crashes caused by motorcyclists running red lights.

Reports of multi-vehicle motorcycle-related crashes occurring at intersections between 2006 and 2010 were extracted for the current research study (see Figure 1 for data inclusion and exclusion). Among 8054 cases relevant to red light violations of motorcycle riders together with non-traffic violation accidents, 2317 (28.7%) involved no injury (property damage only, PDO), 3968 (49.3%) involved minor injury, 931 (11.6%) resulted in serious injury, and 838 (10.4%) resulted in death. A lower proportion of PDO crashes than that of crashes involved in minor injury in the dataset is inconsistent with the fact that the number of crashes decreases with the increase in injury severity [11,12]. Given the potential under-reporting of PDO crashes [13], PDO crashes were excluded from our analysis. Moreover, cases with an absence of motorcycle rider characteristics (e.g., rider age), cases involving foreign riders and cases occurring on expressways were also removed. Thus, 5304 motorcycle-related traffic crashes were selected in the final sample, among which 409 involved red light violations by motorcyclists. The China Road Traffic Accidents Statistics Report showed that the phenomenon of running red lights was very common during the data period. Previous studies have analyzed the red light violations in China that happened in the same period, but with different road users or in different cities (e.g., [2,14,15]). These works provide a comparable foundation for this study to explore the unique factors behind motorcyclists’ RLR behaviours. Therefore, the results arising from the current research on motorcyclists running red lights in Guangdong province of China during 2006 to 2010 have an appropriate level of generality.

In addition, Zhang et al. [2] identified factors influencing red light violations by car drivers, cyclists, and pedestrians from Guangdong province of China. Their results provide a comparative foundation to study the unique factors for motorcyclists’ RLR behaviors from the same province. Therefore, the same data were also used here to study the factors for the severity of car drivers’ injuries in RLR crashes so that the risk factors of severe injuries between motorcyclists and car drivers in RLR crashes could be distinguished.

### 2.2. Risk Factors

The risk factors under consideration in the current study were described in a previous study derived from the same database [2]. Four dimensions, namely personal factors, vehicle factors, road factors and environmental factors, were established as follows.

*Personal factors*: Rider gender, age and occupation are considered to be potential risk factors. Rider age is divided into four categories following the WHO’s age classification criteria: ≤24, 25–44, 45–59, and ≥60 years. Occupation and residential registration are used to capture the education level, income and social status of riders. Rider occupation is divided into six categories: self-employed, worker, migrant worker, farmer, no occupation and other. Residential registration is divided into rural and urban. Additionally, the impact of head injury on the severity of injuries is examined.

*Vehicle factors:* These mainly include whether motorcycles have number plates, whether motorcycles carry passenger(s), vehicle safety conditions and vehicle driving status, where vehicle driving status is divided into four types: going straight, turning left, turning right, and other.

*Road factors:* These mainly include road types, junction types and whether there are physical barriers on the roads. Roads are considered as two types, i.e., general highways (including the first-class and second-class or below highways) and urban roads (including general urban roads and other urban roads). Junctions are divided into three types, i.e., fork, crossroads and other.

*Environmental factors*: Environmental factors include street-light conditions, weather conditions, visibility, weekends, holidays, time periods and years. Street-light conditions include daylight, nighttime with lighting and nighttime without lighting. While bad weather conditions include cloudy, snowy, rainy, foggy, and very windy, poor visibility is defined as visibility below 50 meters. Following the previous work of red light violations [2,8,9], the time period is divided into early morning hours (midnight to dawn), morning peak hours (7:00–8:59 a.m.), after work peak hours (5:00–7:59 p.m.) and other time periods.

### 2.3. Statistical Data Analysis

Binary logit models are widely used in the related literature on motorcycle riders’ RLR behavior at signalized intersections (e.g., [8,9]). To facilitate comparison with the literature using the same method, it was appropriate to adopt binary logit models in this study to estimate the effect of different risk factors on the likelihood of the occurrence of motorcyclists running a red light in China. Specifically, multivariate logistic regression was conducted and the adjusted odds ratios (ORs) of significant factors and their 95% confidence intervals (CIs) were computed using Stata 14 (StataCorp, College Station, TX, USA).

Although motorcycle riders’ red light violations are treated as a serious problem in terms of related injuries and fatalities in developing countries such as China, limited research has considered the analysis of influencing factors for the severity of injuries in motorcycle RLR crashes. Previous injury severity studies have reported a significant correlation among unobserved effects crossing discrete injury outcome categories (e.g., [16]); to further estimate the effect of different risk factors on the likelihood of the occurrence of severe casualties for motorcyclists in RLR crashes in comparison with car drivers, random-parameter logit models were applied in the current research. Random parameters were assumed to be normally distributed and 200 Halton draws were used in this study, which have been widely used assumptions in previous research (e.g., [13,17]).

## 3. Results

### 3.1. Sample Description

Among 5304 motorcycle-related traffic crashes, even though the motorcycle-related crashes caused by RLR are less common (approximately 7.7%) when compared to other causes, they are considered a serious problem. In fact, the ratio of severe injuries among all injuries caused by red light violations of motorcyclists is as high as 44.0%, whereas this ratio for other causes is much smaller, i.e., 38.3% for all motorcycle-related traffic crashes (see Table 1).

### 3.2. Risk Factors Affecting Motorcyclists Running Red Lights

The first model in Table 2 shows the risk factors associated with motorcyclists running red lights. Concerning personal characteristics, the probability of male riders running red lights is 1.48 times greater than that of female riders, which is consistent with conclusions in most of the literature. Compared with motorcyclists over the age of 60, young motorcyclists under the age of 24 (OR = 1.61) shows a significant increase in the probability of running red lights. Chen et al. [8] argued that the young-rider effect could be explained by the fact that young riders, in general, tend to demonstrate risk-taking road behaviors. The risk of running red lights among migrant worker motorcyclists is significantly higher than that among farmers (OR = 1.23), but the impact of residential registration of rider is insignificant.

Among vehicle factors, whether motorcycles carry passengers, whether motorcycles have number plates, vehicle safety conditions and vehicle driving status are significant factors for the probability of motorcyclists running red lights. The probability of running a red light by a motorcyclist riding alone is 1.18 times the probability with passengers. Such results with regard to the presence of a pillion passenger are similar to the study conducted by Chen et al. [8] and Jensupakarn and Kanitpong [9]. The probability of a motorcyclist running a red light is higher when riding a motorcycle that does not satisfy safety requirements (OR = 1.25). However, the probability of a motorcyclist running a red light is lower when riding a motorcycle that does have a number plate (OR = 0.88) As reported in Jensupakarn and Kanitpong’s study [9], the driving direction of a motorcycle also affects whether a motorcyclist runs a red light: compared to driving straight ahead, the probability of a motorcyclist running a red light is significantly higher when they are turning (left: OR = 1.52, right: OR = 1.71, respectively).

Compared with the probability of motorcyclists running red lights on first-class highways, the probabilities of RLR violations by motorcyclists occurring on second-class or below highways, and other urban roads were 1.29 times and 1.23 times higher, respectively. For motorcyclists, the risk of running red lights at forks or crossroads is significantly lower than at other types of intersections. This result differs from the findings of previous studies (e.g., [4]), likely because the surrounding conditions of motorcyclists also influence their likelihood of running red lights. Street lighting can significantly reduce the risk of a motorcyclist running a red light: the risk of running a red light is lower in the daytime (OR = 0.57) and at night with lighting (OR = 0.75) than that at night without lighting. The risk of red light violations by motorcyclists is higher during early morning (OR = 1.27) than during off-peak hours. However, the impact of peak hours on motorcyclists’ red light violations is insignificant, which is inconsistent with the literature [5,8]. Differing from Yan et al. [5], neither weekends nor holidays have significant effects on motorcyclists’ red light violations.

### 3.3. Factors for the Severity of Injuries in Red-Light-Running Crashes

Although motorcyclists are less likely to be involved in RLR traffic accidents than car drivers (7.7% vs. 9.6%), crashes involving motorcyclists running a red light have higher odds of resulting in severe casualties than car drivers (44% vs. 29%) in Guangdong, China; therefore, further comparisons between motorcyclists and car drivers were carried out to examine risk factors related to the severity of outcome in different types of vehicle crashes involving a red light violation. Specifically, random-parameter logit models were conducted for two sub-samples separately, i.e., motorcyclists and car drivers in RLR crashes.

In cases of motorcyclists in RLR crashes, as shown in the second model in Table 2, the estimated parameters for young motorcycle riders were insignificant and random, with a mean of 1.38 and a standard deviation of 35.53. Motorcycle riders registered as residing in rural areas were more likely to get severely injured than those with urban residential registration (OR = 3.21). Furthermore, a motorcyclist’s occupation impacts the level of injury. The probability of serious casualties sustained by the self-employed (OR = 0.19) and workers (OR = 0.14) was significantly lower than that of farmers, but there were no significant differences between the probabilities of migrant workers, the unemployed and those employed in other professions. When a head injury occurs for the motorcycle rider in a RLR crash, the risk of serious casualties increases (OR = 11.80). With regard to the vehicle driving state, a motorcycle rider was more strongly associated with serious casualties when reversing, making a U-turn or changing lanes rather than going straight (OR = 10.24). The estimated parameters for second-class or below highways were insignificant and random, with a mean of 1.35 and a standard deviation of 11.60. Poor visibility can significantly increase the risk of serious casualties in motorcyclists (OR = 3.28), but daylight (OR = 0.22) and bad weather (OR = 0.29) conditions significantly decrease the risk of serious casualties. The risk of serious casualties in motorcyclists is lower during early morning (OR = 0.35) and after work peak hours (OR = 0.16) than during off-peak hours.

By comparing empirical results of the injury models for motorcyclists and car drivers in RLR crashes, this study found that the common risk factor for motorcyclists and car drivers experiencing severe injury in RLR crashes is poor visibility. Moreover, riders/drivers’ residential registration and occupation, vehicle driving status, weather condition, time of a day and whether the rider/driver suffers a head injury are significantly associated with severe casualties in motorcycle crashes related to RLR but have no significant effects on car drivers in RLR crashes.

## 4. Discussion

### 4.1. Red Light Violations and Injury Severity for Motorcyclists

By comparing the influential factors that affect the probability of motorcyclists running red lights and the severity of injuries in crashes caused by red light violations, we found that the common risk factor is daylight condition. Rider gender, rider age, passengers, vehicle number plates, vehicle safety conditions, road type and junction type affect the probability of motorcyclists running red lights but do not affect the severity of injuries. Poor visibility does not affect the likelihood of motorcyclists’ RLR violations, but in the event of RLR crashes, this factor often leads to serious casualties. Notably, the following factors have inconsistent effects on the probability of red light violations and the severity of injuries for motorcycle riders: on the one hand, the likelihood of red light violations when a motorcycle rider is turning left/right is higher than when going straight, but this turning factor has a nonsignificant impact on the severity of injuries; on the other hand, reversing, making a U-turn and changing lanes have nonsignificant effects on the probability of motorcyclists’ RLR violations in contrast to going straight, but have a very significant impact on the severity of injuries. The likelihood of red light violations during the early morning is higher than off-peak hours, but this time factor has a negative impact on the severity of injuries.

### 4.2. Comparison Between Motorcyclists and Other Road Users

Previous studies have reported on the factors contributing to traffic signal violations for car drivers, cyclists and pedestrians in Guangdong, China [2], where the effects of gender have been found to be insignificant for all three groups. In contrast, in this study, male motorcycle riders are confirmed to be more likely to violate traffic signals than female riders. While the likelihood of violating traffic signals during dark conditions is lower than during light conditions for both car drivers and pedestrians, motorcyclists have a significantly increased probability of red light violations during dark conditions. Since travelling on second-class or below highways is a factor contributing to red light violations for car drivers, cyclists, pedestrians and motorcyclists, this factor shall not be treated as a unique factor for motorcyclists running red lights.

For the resulting severe casualties in RLR crashes, as reported in a previous section, poor visibility is a common risk factor for motorcyclists and car drivers experiencing severe injury in RLR crashes; therefore, we can infer that the visibility factor is not unique to motorcyclists with regard to severe injuries related to red light violations.

### 4.3. Policy Implications and Further Remarks

The empirical evidence presented in this article suggests the need for an increase in inspections and punishment for riding a motorcycle with a poor vehicle safety status. In addition, supervision of red light violations should be strengthened during the early morning hours. The lack of regard for and awareness of traffic laws and safety is a major factor affecting behavior related to running red lights. According to the empirical results in this study, males under the age of 24 are the main target group that should be engaged in traffic safety promotion, campaigns, and educational activities.

Due to data availability, the present study analyzed the traffic crash data for 2006–2010 in Guangdong Province, China, which limits its implications, because road safety trends may be different for other provinces in China and may have changed in the same province over the years. Using data from other provinces and cities in China would be of merit in future research. Moreover, a meta-analysis of old and new data from the same province could help us to understand the significant differences in risky behaviors of motorcycle riders in China. Potential factors, such as traffic volume, speed limits, traffic light characteristics and other risky driving behaviors among motorcyclists were not analyzed in this study. It would be worth exploring the effects of these factors on motorcyclists’ RLR violations and injuries in the future.

## 5. Conclusions

Using data collected from Guangdong Province of China, this study offers insights into risk factors related to personal characteristics, vehicle characteristics, road conditions and environmental conditions affecting red light violations and injury severity resulting from motorcyclists’ running red lights. Measures including road safety educational programs for targeted groups and focused enforcement of traffic policy and regulations are suggested to reduce the number of crashes and the severity of injuries resulting from motorcyclists running red lights.

## Figures and Tables

**Table 1:** Descriptive statistics of variables.

Variables	Motorcycle-Related Crashes (*n* = 5304)		Crashes Caused by Red Light Violations of Motorcyclists (*n* = 409)		Crashes Caused by Red Light Violations of Car Drivers (*n* = 435)	
	Frequency	Proportion (%)	Frequency	Proportion (%)	Frequency	Proportion (%)
Signal violation	409	7.7	409	1	435	1
Killed or seriously injured	2031	38.3	180	44.0	126	29.0
(1) Gender						
Male	4776	90.0	374	91.4	409	94.0
(2) Age						
≤24	1133	21.4	81	19.8	47	10.8
25–44	2988	56.3	241	58.9	322	74.0
45–59	982	18.5	76	18.6	65	15.0
≥60	201	3.8	11	2.7	1	0.2
(3) Residential registration						
Rural	1818	34.3	129	31.5	60	13.8
(4) Occupation						
Farmer	1296	24.4	76	18.6	22	5.0
The self-employed	427	8.1	39	9.5	79	18.2
Worker	1053	19.9	85	20.8	74	17.0
Migrant worker	690	13.0	74	18.1	50	11.5
Unemployed	236	4.4	21	5.1	10	2.3
Other occupations	1602	30.2	114	27.9	143	32.9
(5) Whether motorcycles carry a passenger						
No passenger	3576	67.4	273	66.7	279	64.1
(6) Whether motorcycles have number plates						
No number plates	1539	29.0	120	29.3	7	1.6
(7) Vehicle safety condition						
Unfit	394	7.4	17	4.2	16	3.7
(8) Vehicle driving status						
Straight	4524	85.3	326	79.7	319	73.3
Turning left	308	5.8	47	11.5	74	17.0
Turning right	83	1.6	8	2.0	14	3.2
Others	389	7.3	28	6.8	28	6.4
(9) Type of road						
First-class highways	828	15.6	53	13.0	45	10.3
Second-class or below highways	2223	41.9	167	40.8	118	27.1
General urban roads	1663	31.4	164	40.1	208	47.8
Other urban roads	590	11.1	25	6.1	64	14.7
(10) Type of junctions						
Fork	441	8.3	31	7.6	27	6.2
Crossroads	464	8.7	88	21.5	81	18.6
Others	4399	83.0	290	70.9	327	75.2
(11) Whether there are physical barriers in roads						
No physical barriers	3182	60.0	192	46.9	193	44.4
(12) Visibility						
Bad visibility	525	9.9	37	9.0	39	9.0
(13) Street-light condition						
Daylight	2938	55.4	218	53.3	212	48.7
Dark but lighted	1527	28.8	167	40.8	204	46.9
Dark	839	15.8	24	5.9	19	4.4
(14) Weather condition						
Bad weather condition	1089	20.5	71	17.4	98	22.5
(15) Holiday						
Holiday	381	7.2	30	7.3	31	7.1
(16) Day of the week						
Weekends	1401	26.4	109	26.7	135	31.0
(17) Time of day						
Early morning	804	15.2	64	15.6	98	22.5
Morning peak hours	712	13.4	51	12.5	48	11.0
After work peak hours	911	17.2	63	15.4	58	13.3
Others	2877	54.2	231	56.5	231	53.1
(18) Year						
2006	922	17.4	108	26.4	107	24.6
2007	960	18.1	64	15.6	74	17.1
2008	1075	20.3	71	17.4	75	17.2
2009	1127	21.2	85	20.8	78	17.9
2010	1220	23.0	81	19.8	101	23.2
(19) Injured parts						
Head	1390	26.2	101	24.7	15	3.4

**Table 2:** Factors influencing motorcyclists running red lights and resulting severe casualties.

Factors	Red Light Violations for Motorcyclists	Severe Casualties for Motorcyclists in Red-Light-Running Crashes	Severe Casualties for Car Drivers in Red-Light-Running Crashes
	ORs (95% CI)	ORs (*s.d.*) (95% CI)	ORs (*s.d.*) (95% CI)
*n*	5304	409	435
(1) Personal Factors			
Gender of rider (base: female)			
Male	1.48 ***		0.17 **
	[1.20, 1.82]		[0.04, 0.70]
Age of rider (base: ≥60)			
≤24	1.61***	1.38 (35.53 *)	7.11 **
	[1.15, 2.26]	[0.16, 12.11]	[1.53, 33.05]
25–44			2.79 *
			[0.92, 8.45]
Residential registration of rider (base: urban)			
Rural		3.21 *	
		[0.87, 11.80]	
Occupation of rider (base: farmer)			
The self-employed		0.19 *	
		[0.03, 1.36]	
Migrant worker	1.23 *		
	[1, 1.52]		
Worker		0.14 **	
		[0.02, 0.97]	
Injured parts (base: others)			
Head	NA	11.80 ***	
		[3.15, 44.15]	
(2) Vehicle factors			
Carry passenger (base: yes)			
No passenger	1.18 **		0.33 ***
	[1.04, 1.34]		[0.16, 0.71]
Whether motorcycles have number plates (base: yes)			
No number plates	0.88 *		
	[0.77, 1.01]		
Vehicle safety condition (base: fit)			
Unfit	1.25 **		
	[1.01, 1.56]		
Vehicle driving status (base: straight)			
Turning left	1.52 ***		
	[1.19, 1.93]		
Turning right	1.71 **		
	[1.09, 2.68]		
Others		10.24 ***	
		[2.33, 44.95]	
(3) Road factors			
Type of road (base: first-class highways)			
Second-class or below highways	1.29 ***	1.35 (11.60*)	6.06 **
	[1.07, 1.54]	[0.40, 4.55]	[1.41, 26.12]
General urban roads			1.22
			[0.33, 4.59]
Other urban roads	1.23 *		1.54
	[0.97, 1.56]		[0.33, 7.26]
Type of junction (base: others)			
Fork	0.62 ***		
	[0.50, 0.78]		
Crossroads	0.62 ***		
	[0.50, 0.78]		
(4) Environmental factors			
Visibility (base: others)			
Poor visibility		3.28 *	3.15 **
		[0.91, 11.87]	[1.14, 8.66]
Street-light condition (base: dark)			
Daylight	0.57 ***	0.22 *	0.85 (73.51 **)
	[0.48, 0.69]	[0.04, 1.15]	[0.09, 8.31]
Dark but lighted	0.75 ***		2.45
	[0.62, 0.91]		[0.54, 11.15]
Weather condition (base: others)			
Bad weather condition		0.29 **	
		[0.09, 0.92]	
Holiday (base: others)			
Holiday			0.13 *
			[0.02, 1.04]
Time of day (base: others)			
Early morning	1.27 ***	0.35 *	
	[1.07, 1.51]	[0.11, 1.12]	
Morning peak hours			
After work peak hours		0.16 ***	
		[0.04, 0.62]	
log likelihood	−3371.41	−219.13	−214.66
AIC	6814.83	516.27	511.31

For brevity, insignificant results are omitted, and standard deviations are presented for significant random variables only. * *p* < 0.1, ** *p* < 0.05, *** *p* < 0.01.

## Data Availability

No new data were created or analyzed in this study. Data sharing is not applicable to this article.
